# Diabetic Risk Factors Promote Islet Amyloid Polypeptide Misfolding by a Common, Membrane-mediated Mechanism

**DOI:** 10.1038/srep31094

**Published:** 2016-08-17

**Authors:** Alan K. Okada, Kazuki Teranishi, J. Mario Isas, Sahar Bedrood, Robert H. Chow, Ralf Langen

**Affiliations:** 1 Department of Biochemistry and Molecular Biology, Zilkha Neurogenetic Institute, University of Southern California, Los Angeles, California, USA; 2 USC Roski Eye Institute, Department of Ophthalmology, Keck School of Medicine, University of Southern California, Los Angeles, California, USA; 3 Department of Physiology and Biophysics, Keck School of Medicine, Zilkha Neurogenetic Institute, University of Southern California, Los Angeles, California, USA

## Abstract

The current diabetes epidemic is associated with a diverse set of risk factors including obesity and exposure to plastics. Notably, significant elevations of negatively charged amphiphilic molecules are observed in obesity (e.g. free fatty acids and phosphatidic acid) and plastics exposure (monophthalate esters). It remains unclear whether these factors share pathogenic mechanisms and whether links exist with islet amyloid polypeptide (IAPP) misfolding, a process central to β-cell dysfunction and death. Using a combination of fluorescence, circular dichroism and electron microscopy, we show that phosphatidic acid, oleic acid, and the phthalate metabolite MBzP partition into neutral membranes and enhance IAPP misfolding. The elevation of negative charge density caused by the presence of the risk factor molecules stabilizes a common membrane-bound α-helical intermediate that, in turn, facilitates IAPP misfolding. This shared mechanism points to a critical role for the membrane-bound intermediate in disease pathogenesis, making it a potential target for therapeutic intervention.

The misfolding and aggregation of the 37-amino acid peptide, islet amyloid polypeptide (IAPP), is thought to be one of the main factors responsible for the β-cell defect that drives the pathogenesis of type 2 diabetes mellitus (T2DM). More than 90% of patients with T2DM have IAPP aggregates, and misfolded IAPP is toxic to cell lines. Transgenic animals expressing the aggregation-prone human IAPP develop T2DM symptoms^1^,^2^,^3^,^4^. Epidemiological studies show that the growing diabetes epidemic is correlated with an epidemic of obesity/metabolic syndrome, and to exposure to plastics containing phthalates, a family of plastic additives known as plasticizers. Whether metabolic or phthalate-based risk factors act independently of IAPP or exert their effects by affecting IAPP misfolding is unknown.

Interestingly, both plasticizer exposure and metabolic syndrome lead to significant elevations of negatively charged amphiphilic lipid or lipid-like molecules (Fig. S1). For example, rodent models of obesity have shown that levels of the negatively charged lipid, phosphatidic acid (PA), are elevated in the insulin- and IAPP-producing pancreatic β-cells^5^,^6^. Moreover, insulin resistance, obesity and dyslipidemia result in remarkably high levels of free fatty acids (FFAs) that can reach up to millimolar concentrations^7^,^8^,^9^,^10^. On the other hand, exposure to certain everyday plastics leads to elevated blood and urine levels of negatively charged phthalate metabolites, known to be associated with a decrease in β-cell function and T2DM^11^,^12^,^13^,^14^. These amphiphilic, negatively charged molecules are generated as metabolic by-products of phthalate-based plasticizers, which are added to plastics in order to increase flexibility. Exposure to plasticizers occurs under many different circumstances, as plasticizers leach out of many common materials. For phthalate-based plasticizers, these include food packaging, furniture, toys, medical devices, cosmetics, perfumes, lotions, paints, lacquers, varnishes, and pharmaceuticals^11^. Thus, a better understanding of their potential toxic effects is needed.

The central question addressed in the present study is whether the aforementioned, negatively charged lipids or lipid-like molecules promote IAPP misfolding. If so, this would indicate that metabolic and phthalate-based risk factors could contribute to T2DM pathogenesis by enhancing IAPP toxicity. Prior work has shown that negatively charged membranes containing phosphatidylserine or phosphatidylglycerol can promote IAPP misfolding^15^,^16^. This membrane-mediated misfolding pathway involves an attractive interaction between the positively charged IAPP and the negatively charged membrane surface, which results in a transient α-helical structure of IAPP that catalyzes misfolding. Here we wanted to test whether PA, FFAs and phthalate metabolites (monophthalate esters) might similarly be able to facilitate misfolding via a membrane-mediated misfolding pathway. While the lipid PA is naturally found in membranes, FFAs and monophthalate esters will insert their hydrophobic regions into phospholipid membranes, leaving their negatively charged carboxyl groups at the membrane surface. By embedding into the membrane, these molecules would enhance the negative charge density at the membrane surface and thereby promote IAPP misfolding.

In order to address this question, we chose oleic acid (OA) as an example for a FFA, and we used monobenzyl ester phthalate, MBzP, as an example of a monophthalate ester (Fig. S1). OA was chosen because it is the most abundant FFA in pancreatic fat and in serum. Importantly, mice fed on a high fat diet show significant increases in pancreatic free-OA and triacylglycerol–associated OA. Furthermore, patients with higher pancreatic fat show a similar trend^17^,^18^. MBzP was of particular interest because it is known to be associated with T2DM risk^11^,^12^,^13^,^14^. Our data indicate that OA and MBzP strongly partition into membranes. Using circular dichroism (CD), fluorescence spectroscopy and transmission electron microscopy (EM) we also found that physiologically relevant amounts of PA, OA and MBzP potently promote IAPP misfolding via a membrane-bound α-helical intermediate. Collectively, these data directly link T2DM risk factors to IAPP misfolding via a common catalytic pathway.

## Results

### Phosphatidic Acid-LUVs accelerate IAPP misfolding

**Figure 1:**
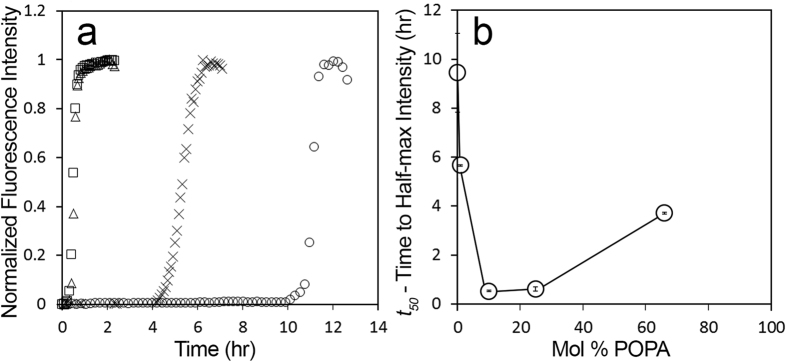
Phosphatidic acid modulates the rate of IAPP fibrilization. IAPP misfolding was measured as a function of time using ThT and demonstrates a dependence upon the mol% of PA. (**a**) Representative ThT curves from experiments with 12.5 μM human IAPP and 500μM lipid in 10mM phosphate buffer pH 7.4 during incubation with LUVs composed of 66% PA (omitted in A), 25% PA (□), 10% PA (Δ), 1% PA (X), and 0% PA (100% PC, ○). B) Comparison of average *t*_*50*_ values with the mol% PA from experiments in A. Error bars represent one standard deviation from a minimum of 3 experiments per condition. (*p* < *0.05 for 1%, and p* < *0.01 for all other conditions, compared with 100% PC-LUV control*).

In order to test whether the negatively charged phospholipid PA can modulate IAPP misfolding via a membrane-mediated misfolding mechanism, we used thioflavin T (ThT) fluorescence. IAPP was incubated with PA-LUVs, large unilamellar vesicles (LUVs) composed of varying ratios of phosphatidic acid (PA) and the neutral phospholipid phosphatidylcholine (PC). The kinetics of IAPP misfolding were quantified by determining the time to half-maximal fluorescence (*t*_*50*_) using a sigmoidal fit, an example of which is given in Supplemental Fig. S2. IAPP achieved half-maximal fluorescence (*t*_*50*_) after approximately 9.5 h in the presence of PC-only vesicles (i.e. 100% PC-LUV) (Fig. 1b). All vesicles containing PA accelerated IAPP misfolding. Remarkably, even 1 mol% PA, which corresponds to a substoichiometric concentration of PA molecules (1 PA:2.5 IAPP), sped up misfolding by almost 4 hours (Fig. 1). The strongest acceleration of misfolding was observed for 10 and 25% PA where misfolding occurred nearly 20 times faster than in the control. This robust acceleration was mildly attenuated in the presence of 66 mol% PA. Overall, the behavior is reminiscent of what we observed for negatively charged PS membranes, where the maximal enhancement of misfolding was observed at 25% PS and where higher amounts of PS progressively reduced the kinetics of misfolding^15^.

Interactions between the negatively charged PA and the positively charged IAPP are likely electrostatic in nature^19^. To test this notion, we examined the effect of increased ionic strength on misfolding by repeating the experiments of Fig. 1 in the presence of salt (100 mM NaCl). We found that PA-LUVs retain their ability to accelerate IAPP misfolding under these conditions, but that the optimum acceleration of misfolding occurs at a higher percentage of PA (Figs S3 and S4). A similar effect was observed when IAPP misfolding was triggered by PS^15^. These data suggest that, just as in the case of PS membranes, salt shields the electrostatic interactions between the negative membrane and the positive peptide.

### IAPP transiently adopts an α-helical conformation with PA-LUVs before transitioning to a β-sheet rich structure

**Figure 2:**
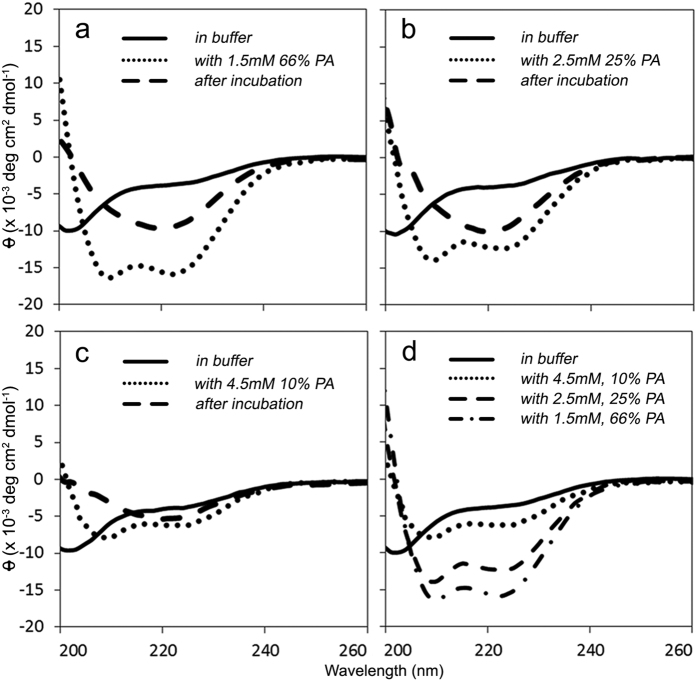
IAPP forms an α-helix upon interaction with phosphatidic acid containing LUVs before transitioning into a β-sheet structure. (**a**–**c**) Representative circular dichroism spectra are shown. The secondary structure of 25 μM IAPP was first determined in 10 mM phosphate buffer, pH 7.4 (**—**). (**a**) minimum at 202 nm suggests a mostly disordered structure. LUVs composed of 66 (**a**), 25 (**b**), and 10 mol% (c) POPA were added and the structure was immediately measured (•••). Minima at 208 nm and 222 nm indicate a transition into an α-helix upon interaction with LUVs. After incubation at room temperature, the samples were measured a final time (**— —**). A minimum near 218 nm is most consistent with a β-sheet structure. D) Initial spectra of IAPP upon addition of 66 (**— • —**), 25 (**— —**), and 10 mol% (•••) PA-LUVs are overlaid with the disordered structure in buffer (**—**), provided for reference.

We next set out to determine what structural changes occur as IAPP misfolds in the presence of PA-LUVs. IAPP secondary structure was determined by CD at different time points throughout the misfolding process. These experiments were limited to the low salt conditions as chloride absorbs strongly in the lower UV range, which interferes with CD measurements. Immediately after dissolution in buffer, IAPP displayed a negative peak near 202 nm (Fig. 2a–d, solid line), characteristic of a mostly disordered structure. Upon addition of PA-LUVs, IAPP immediately transitioned into an α-helical structure as indicated by the negative peaks at 208 and 222 nm (Fig. 2a–c, dotted line). Within minutes to hours (depending upon conditions) the helical structure then transitioned into a β-sheet rich conformation, indicated by the negative peak near 218 nm (Fig. 2a–c, dashed line). The initial degree of helicity increased with the PA content of the vesicles (Fig. 2d). Using previously established methods^20^,^21^, we estimated the percentage of helicity for IAPP in the presence of 66 mol% PA-LUVs to be approximately 45%. Given that IAPP is 37 residues in length and assuming full binding, this estimate corresponds to ~17 amino acids participating in the membrane-bound helical structure. These findings are in line with prior estimates from IAPP bound to membranes containing high percentages of PS^15^.

### Electron microscopy of IAPP incubated with PA-LUVs

**Figure 3:**
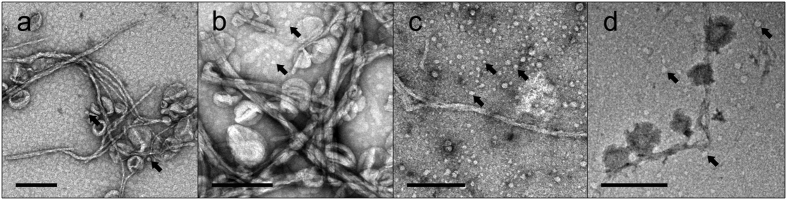
Electron microscopy reveals IAPP fibrils in some cases decorated with PA-LUVs. 12.5 μM IAPP was allowed to misfold in the presence of 500 μM PA-containing LUVs. Samples were incubated for the amount of time required to achieve ThT positivity, as in Fig. 1, before being applied to the micrograph grid. (**a**–**d**) EM micrographs of IAPP in 10 mM phosphate buffer, pH 7.4 with 66 (**a**), 25 (**b**), 10 (**c**), and 1 mol% (**d**) PA-LUVs. These images show fibrils, many of which are decorated with LUVs. In some cases the shape of the LUVs are distorted where they contact fibrils. Lipid free sections of fibrils measure diameters of ~7 nm. Some fibrils assemble laterally, increasing the apparent fibril diameter. Arrows indicate small spherical structures 6 nm in diameter and greater (arrows). Scale bars = 200 nm.

Collectively, the enhanced ThT fluorescence and β-sheet formation strongly suggested the formation of IAPP fibrils. For additional validation, we visualized IAPP by negative-stain transmission electron microscopy (EM) at late time points when the ThT readings had plateaued (Fig. 3). Indeed, fibrils could be observed in the EM. The morphology of fibrils formed in the presence of PA-LUV was similar to those seen in the absence of lipids. Both cases yielded long, non-branching fibrils that were often ribbon-like, sometimes twisted, and had diameters of ~7 nm. In some areas, it appeared as if lateral assemblies of individual fibril strands thickened the apparent diameter of fibrils. Interestingly, where lipids are present and contacting the surface of the fibrils, these lipids are often distorted and also contribute to an apparent thickening of the fibril diameter as in Fig. 3. In nearly all cases, fibrils can be found free of PA-LUVs. In addition, small rounded structures were formed when IAPP and PA-LUVs were incubated. Typically their sizes ranged from ~6 to 20 nm in diameter. These structures could represent oligomeric IAPP, small protein-lipid particles as was observed for a-synuclein^22^,^23^ or both.

Taken together these data suggested that PA can accelerate misfolding in a manner similar to PS, further supporting the notion that negative membrane charge, rather than the specific nature of the lipids, plays a key role in facilitating membrane-mediated misfolding.

### Oleic Acid-LUVs accelerate IAPP misfolding kinetics

**Figure 4:**
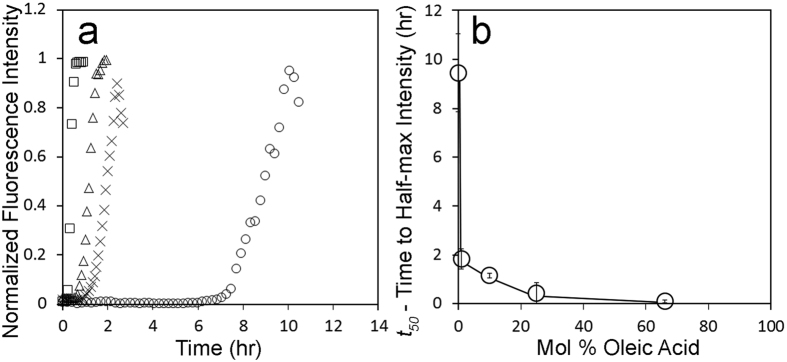
Oleic Acid enhances IAPP misfolding kinetics. ThT was used to follow IAPP aggregation kinetics. (**a**) IAPP aggregation was measured as a function of time using ThT and demonstrates dependence upon the mol% of oleic acid. (**a**,**b**) Representative ThT curves from experiments with 12.5 μM human IAPP and 500 μM oleic acid in 10 mM phosphate buffer pH 7.4 25% (□), 10% (Δ), 1% oleic acid (x), and 100% POPC (○). (**b**) Comparison of *t*_*50*_ values with the mol% of oleic acid in LUVs from experiments in (**a**). Error bars represent one standard deviation from a minimum of 3 experiments per condition. (*p* < *0.01 for all conditions compared to 100% POPC LUV control*).

To test whether membrane-embedded amphiphilic molecules such as fatty acids can mimic negatively charged lipids and enhance IAPP misfolding kinetics, we measured the ability of PC-based LUVs containing the fatty acid, oleic acid (OA-LUV)^18^,^24^, to affect IAPP misfolding (Fig. 4). Using ThT fluorescence, we monitored IAPP misfolding in the presence of OA-LUVs containing 1, 10, 25, and 66 mol% oleic acid. Compared to PA and PS^15^, OA enhanced misfolding even more. Particularly remarkable was the finding that even small substoichiometric amounts of 1% OA (1 OA: 2.5 IAPP) reduced the *t*_*50*_ by nearly 7 hours. The fastest kinetics were obtained for 66 mol% OA, where misfolding was enhanced by more than 100 fold. Given that OA strongly partitions into PC membranes^25^,^26^, the enhanced misfolding was expected to be caused by membrane-embedded, rather than soluble OA. Inasmuch as soluble OA has the capacity to modulate IAPP misfolding^27^, we further tested this notion using centrifugation of OA-LUVs. The supernatant, which would contain any soluble OA, did not cause acceleration of IAPP misfolding kinetics and did not change IAPP secondary structure according to CD (Fig. S5). Taken together, these data show a robust and significant enhancement of IAPP misfolding kinetics by all OA-LUV mol%’s tested that occurs at or on LUV membranes.

### IAPP transiently adopts α-helical conformation with OA-LUVs before transitioning into β-sheet structure

**Figure 5:**
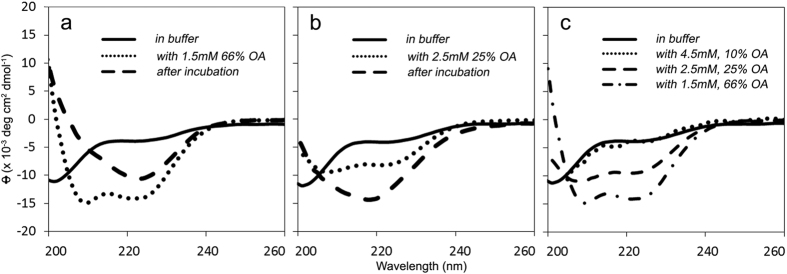
IAPP forms an α-helix upon interaction with oleic acid containing LUVs before transitioning into a β-sheet structure. Representative circular dichroism spectra are shown. The secondary structure of 25 μM IAPP was first determined in 10 mM phosphate buffer, pH 7.4 (solid line). A minimum at 202 nm suggests a mostly disordered structure. LUVs composed of 66 (**a**) and 25 mol% (**b**) oleic acid were added and the structure was measured again (dotted line). Minima at 208 nm and 222 nm indicate a transition into an α-helix upon interaction with LUVs. After incubation at room temperature, the samples were measured a final time (dashed line). A minimum near ~218 nm is most consistent with a primarily β-sheet structure. (**c**) Initial spectra of IAPP upon addition of 66 (**— • —**), 25 (**— —**), and 10 mol% (**•••**) OA-LUVs are overlaid with the disordered structure in buffer (**—**), provided for reference.

We next tested whether the acceleration of IAPP misfolding by OA-LUVs also proceeded via a helical intermediate. We used CD to monitor changes in IAPP secondary structure during incubation with 10, 25 and 66 mol% OA-LUVs (Fig. 5). At initial time points immediately after mixing, we observed an increase in helicity with increasing percentage of OA (Fig. 5c). Over time, helical IAPP transitioned into a β-sheet-rich structure, as indicated by the single negative peak between 218–220 nm (Fig. 5a,b).

### Electron microscopy of IAPP incubated with OA-LUVs

**Figure 6:**
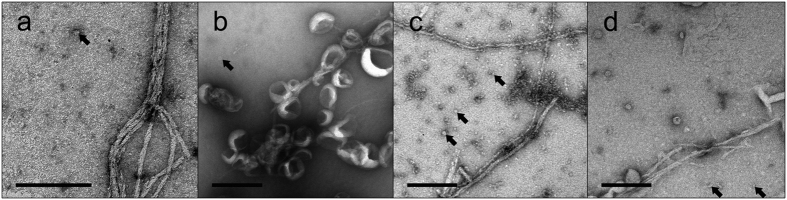
Electron microscopy reveals OA-LUV mediated misfolding of IAPP yields IAPP fibrils in some cases decorated with OA-LUVs. 12.5 μM IAPP was allowed to misfold in the presence of 500 μM OA-LUVs. Samples were incubated for the amount of time required to achieve ThT positivity, as in Fig. 1, before being applied to the micrograph grid. (**a**–**d**) EM micrographs of IAPP in 10 mM phosphate buffer, pH 7.4 with 66 (**a**), 25 (**b**), 10 (**c**), and 1 mol% (**d**) OA-LUVs show fibrils decorated with LUVs, in some cases the shape of the LUVs are distorted where they contact fibrils. Lipid free sections of fibrils measure diameters of ~7 nm. In many cases lateral assemblies of fibrils are evident [as are small spherical structures 5 nm in diameter (arrows)]. Scale bars = 200 nm.

Negative-stain electron microscopy verified the formation of fibrils in the presence of OA-LUVs (Fig. 6). As in the aforementioned cases, fibrils were frequently in contact with vesicles. Distortion of the vesicles can sometimes be observed and in those instances contributes to an apparent widening of fibril diameters. Examples of fibrils not in contact with LUVs can be found in all conditions. Again, small rounded structures could also be observed under these conditions. Collectively, these data are very similar to those obtained for PA-LUVs and show that OA can promote membrane-mediated misfolding in a manner akin to negatively charged phospholipids.

### Monobenzyl ester phthalate-LUVs accelerate IAPP misfolding kinetics

**Figure 7:**
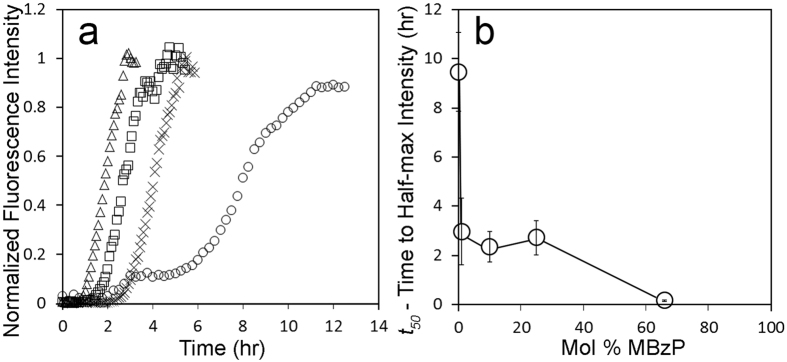
Monobenzyl ester phthalate-LUVs enhance IAPP misfolding kinetics. ThT was used to follow IAPP aggregation kinetics. (**a**) IAPP aggregation was measured as a function of time and demonstrates dependence upon the mol% of MBzP. (**a**) Representative ThT curves from experiments with 12.5 μM human IAPP and 500 μM MBzP-LUVs in 10 mM phosphate buffer pH 7.4 demonstrate acceleration of fibrilization for 66 (omitted in A), 25 (□), 10 (Δ), 1 mol% MBzP (x), and 100% POPC (○). (**b**) Comparison of *t*_*50*_ values from experiments in A. Error bars represent one standard deviation from a minimum of 3 experiments per condition. (*p* < *0.01, for all conditions compared with 100% POPC LUV control*).

Next we tested whether another negatively charged, amphiphilic molecule, the monophthalate ester, monobenzyl ester phthalate (MBzP), could similarly mimic negatively charged lipids and promote IAPP misfolding. Toward this end, we used ThT fluorescence to measure the misfolding kinetics of IAPP in the presence of PC-based MBzP-LUVs (Fig. 7). MBzP-LUVs significantly enhanced the rate of IAPP misfolding. Again, we used centrifugation in order to verify that the effects were caused by membrane-bound rather than soluble MBzP. As in the case of OA-LUVs, we found that the vesicle fraction, but not the supernatant, was capable of enhancing aggregation (Fig. S5). These data indicated that MBzP strongly partitioned into the bilayer, however, as MBzP partitioning into PC bilayers had not been described in the literature, we determined the partitioning of MBzP into PC membranes. First, we determined that the extinction coefficient for MBzP at 237 nm is 6633.2 M^−1^cm^−1^ (see materials and methods). We used this extinction coefficient in order to quantify the amounts of MBzP free in solution and bound to the vesicles, and arrived at a partitioning coefficient of 1.1 ± 0.7 × 10^−5^ (for details see materials and methods). This coefficient further demonstrated that the concentration of MBzP in solution relative to that in the membrane is very low. Unfortunately, the strong UV absorption of MBzP-LUVs prohibited us from using CD to determine the secondary structure of IAPP within the MBzP-LUV membranes.

### Electron microscopy of IAPP incubated with MBzP-LUVs

**Figure 8:**
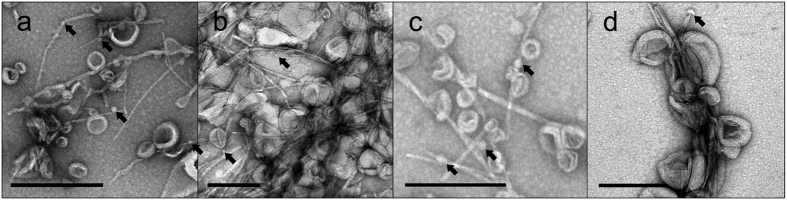
Electron microscopy reveals IAPP fibrils in some cases decorated with MBzP-LUVs. 12.5 μM IAPP was allowed to misfold in the presence of 500 μM PA-containing LUVs. Samples were incubated for the amount of time required to achieve ThT positivity, as in Fig. 1, before being applied to the micrograph grid. (**a**–**d**) EM micrographs of IAPP in 10 mM phosphate buffer, pH 7.4 with 66 (**a**), 25 (**b**), 10 (**c**), and 1 mol% (**d**) MBzP-LUVs show fibrils decorated with LUVs. Some LUVs are distorted where they contact fibrils. Lipid free sections of fibrils measure diameters of ~7 nm. In many cases lateral assemblies of fibrils are evident as are small spherical structures 10 nm in diameter and greater (arrows). Scale bars = 200 nm.

We used negative-stain EM to visualize IAPP misfolding end-products following incubation with MBzP-LUVs. Applying the same methodology as before (see Figs 3 and 6), we observed that all lipid conditions produced IAPP aggregates, the primary constituent of which were fibrils (Fig. 8). Furthermore, those fibrils were often physically associated with MBzP-LUVs. Where this association occurs, distortion of the vesicle morphology can often be observed. Again, small rounded structures were sometimes present. Collectively, these data demonstrate that MBzP partitions strongly into membranes and enhances IAPP misfolding.

## Discussion

The current T2DM epidemic is fueled by a number of risk factors, making it important to understand how these risk factors contribute to disease pathogenesis. Here we specifically investigate three negatively charged, amphiphilic molecules associated with known metabolic and phthalate-based risk factors. All of these molecules are currently considered to act independently of IAPP misfolding. Metabolic abnormalities and obesity in particular are thought to drive T2DM by promoting insulin resistance. In addition, metabolic dysfunction can lead to a heightened inflammatory state that may also contribute to T2DM^27^. Phthalate-based risk factors are hypothesized to contribute to T2DM by altering fatty acid metabolism, lipid homeostasis and adipogenesis via peroxisome proliferator-activated receptor (PPAR) activation^28^,^29^. Our data indicate, however, that PA, OA and MBzP all have a pronounced effect on IAPP misfolding. All three molecules partition into membranes where they enhance negative charge density and promote membrane-mediated misfolding. These data strongly suggest that the risk factors can also contribute to T2DM pathogenesis through an IAPP-dependent pathway.

The effects on IAPP misfolding required remarkably small amounts of negatively charged molecules. In all cases, substoichiometric amounts were sufficient to strongly catalyze misfolding. The most pronounced effects were observed for OA-containing membranes, where the addition of 1% OA increased misfolding more than 4 fold. Thus, even relatively small amounts of these molecules can significantly affect IAPP misfolding. The emerging picture from protein misfolding or amyloid diseases is that factors that promote misfolding enhance the likelihood of disease. In contrast, inhibition of misfolding^30^,^31^, enhanced clearance or degradation of misfolded proteins via heat shock proteins, proteasome or autophagy pathways tend to reduce pathology^32^. Typically, protein misfolding diseases are late in onset and it often takes years for imbalances in protein homeostasis to lead to clinical symptoms. We therefore envisage a model in which the three classes of molecules tested here adversely effect protein homeostasis by continually favoring the misfolded state(s) of IAPP and ultimately cause disease.

How physiologically relevant are the concentrations used in our study? The concentrations for PA have recently been reported in rodent obesity models. In these studies, the PA levels in membranes were found to be in the range of ~3 to 5% mol%, while non-obese rodents had less than 2% PA and cardiolipin combined^5^. As shown in Fig. 1, these percentages are in a range where significant enhancements of IAPP misfolding can be observed. The precise concentrations of MBzP and OA in cellular membranes are not known, but we can estimate that the concentrations of these molecules can also likely reach the range tested in the present study. The strong partitioning of MBzP into PC membranes suggests that significant amounts of monophthalate ester must localize to cellular membranes. MBzP serum concentrations of ~0.14 μM^33^ have been reported. Based on the *K*_*p*_ determined in the present study, mol percentages of 1% MBzP could easily be accomplished in membranes. As mentioned above, obesity can drive FA concentrations into the millimolar range^8^,^9^,^10^,^34^,^35^,^36^ and the concentrations are potentially even higher in the local environment of the fatty pancreas^7^,^24^,^37^,^38^,^39^,^40^,^41^. Regardless of the precise number it is clear that the FA concentrations are orders of magnitude higher than those of MBzP and small percentages of FA, similar to those tested here, could readily accumulate in cellular membranes.

Our data also indicate that the enhancement of membrane-mediated misfolding of IAPP is a rather generic property of membranes that largely depends upon its negative charge density and to a lesser extent on the exact chemical nature of the negatively charged lipid. In fact, this property does not even require bona fide phospholipids and can be mimicked by negatively charged amphiphilic molecules that can partition into the membrane. Moreover, in all cases tested misfolding proceeded via an α-helical intermediate. This helical intermediate has been suggested to anchor IAPP into the membrane where two-dimensional diffusion can rapidly promote intermolecular interactions in a low dielectric environment that promotes secondary structure formation, including misfolding to β-sheet structure^19^,^42^. Preventing the membrane-bound helical structure from forming or stabilizing it to prevent further misfolding would be beneficial in counteracting the effects of the risk factors driving high T2DM incidence rates. In fact, recent work has shown that IAPP-helix stabilizing/targeting agents can prevent IAPP membraned-mediated misfolding and cytotoxicity^43^,^44^,^45^. Based on our data, we suspect that this approach represents a promising avenue to mitigate the harm caused by risk factors like obesity and phthalate exposure. Finally, membrane-mediated misfolding has been observed for amyloid-β and α-synuclein^46^. Thus, it may well be possible that analogous risk factors may also exist that promote membrane-mediated misfolding in other amyloid diseases.

## Materials and Methods

### Materials

Hexafluoro-2-propanol (HFIP), and Oleic Acid (OA) were obtained from Sigma-Aldrich (St. Louis, MO). Thioflavin T (ThT) was obtained from Sigma-Aldrich (Milwaukee, WI). 1-Palmitoyl-2-oleoyl-sn-glycero-3-phosphatidylcholine (POPC) and 1-palmitoyl-2-oleoyl-*sn*-glycero-3-phosphate (POPA) were obtained from Avanti Polar Lipids (Alabaster, AL) as stocks concentrated in chloroform. Monobenzyl ester phthalate (MBzP) was obtained from Tokyo Chemical Industry - America (Portland, OR). Synthetic wild-type human IAPP was obtained from Bachem (Torrance, CA).

### Preparation of IAPP

IAPP was received from Bachem in a lyophilized powder, dissolved in HFIP, aliquoted into individual tubes, frozen in N_2_ (*l*) and lyophilized. Protein concentrations were determined by UV absorbance at 280 nm in 8 M guanidinium chloride using an extinction coefficient of 1405 M^−1^cm^−1^ and verified by CD spectroscopy upon resolublization. Lyophilized IAPP stocks were stored, desiccated at −80 °C.

### Preparation of Large Unilamellar Vesicles (LUV)

Lipid vesicles were made by combining the indicated molar ratios of PC with either PA, OA or MBzP dissolved in chloroform or ethanol, evaporated to a dry film with N_2_ (*g*) and vacuum desiccated overnight. All PC was of the POPC type while all PA was of the POPA type. The dried lipids were reconstituted in the appropriate buffer and freeze-thawed at least 6 times before extrusion through a mini-extruder containing a polycarbonate filter to a diameter of 100 nm. For OA- and MBzP- LUVs, a centrifugation step (55,000*g for 30 minutes) was added prior to extrusion to eliminate potential micellar contaminants. The supernatant was removed following centrifugation and replaced with appropriate buffer.

### Thioflavin T Fluorescence Assay

A 5 mM stock concentration of Thioflavin T (ThT) was stored at −20 °C in water and used at a 25 μM final concentration as before to monitor IAPP misfolding kinetics^15^. IAPP aliquots were prepared as above. Individual samples of IAPP were solubilized in appropriate buffer with ThT to an appropriate concentration from a dry powder. Prior to the addition of lipid, the solution was loaded into a 2 mm path length quartz cuvette. CD and ThT readings were taken to verify the concentration and quality of the IAPP. At time = 0, LUVs were added, the solution mixed, and place into a JASCO FP-6500 fluorometer. Fluorescence was monitored under the following settings and conditions: excitation wavelength = 450 nm, emission wavelength = 482 nm, excitation slit width = 1 nm, emission slit width = 10 nm, temperature = room temperature, and pH = 7.4.

*t*_*50*_ values were determined as before^15^. Briefly, ThT fluorescence emission intensity was recorded as a function of time. The data was fitted to a sigmoidal curve given by equation (1)


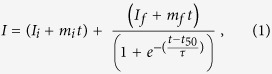


where *I* is the ThT fluorescence intensity, *t* is time, and *t*_*50*_ is time at half-maximal fluorescence intensity. All other parameters are as described previously^47^. *t*_*50*_ values were obtained from the fit to our data and used to compare misfolding kinetics of the various experimental conditions. Normalization was performed by dividing the fluorescence intensity by a maximum intensity value read following the end of each experiment. Maximum intensity values are determined in the following way. After a plateau phase is observed, the cuvette is removed, shaken and re-measured to control for any potential settling artifacts. This process is repeated as needed to confirm a plateau phase has been reached. If a settling artifact disrupts the growth phase, the *t*_*50*_ becomes difficult to ascertain and such data cannot be used.

### Circular Dichroism Spectroscopy

IAPP was prepared as above. Individual aliquots were solubilized in appropriate buffer and transferred to a 1 or 2 mm quartz cuvette. CD spectra were measured between 195 and 260 nm in a JASCO 815 spectropolarimeter. Measurements were taken every 1 nm at a scan rate of 50 nm/min, with an averaging time of 1 s and background subtracted using appropriate backgrounds.

The fraction of helicity was determined using the previously determined relationships^20^,^21^ described in equations (2) and (3)


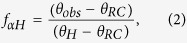


where *f*_*αH*_ is the fraction of helicity, *θ*_*obs*_ is the observed ellipticity, *θ*_*RC*_ is the ellipticity value for a fully random coiled peptide equal in length to IAPP, and *θ*_*H*_ is the ellipticity value for a fully helical peptide equal in length to IAPP. Ellipticity values were taken at 222 nm and converted into mean residual ellipticity using equation (3)


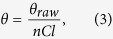


where *θ* is the mean residual ellipticity (deg, cm^2^ dmol^−1^), *θ*_*raw*_ is the ellipticity in millidegrees, *n* is the number of amino acids, *C* is the molar concentration, and *l* is the path length in millimeters. The relationship described by equation (2) is only valid for proteins passing through a helix-coil transition in conditions of constant temperature. Our experiments satisfy these conditions. In addition, the accuracy of the estimate generated by this equation depends in part on the values used to represent fully helical or random coil structures. As in our previous study, we used a value of *θ*_*H*_ = −34.7 × 10^3^ deg cm^2^ dmol^−1^ and *θ*_*RC*_ = −1.5 × 10^3^ deg cm^2^ dmol^−1^ to describe fully helical and random coil structures, respectively^15^. Finally, despite using excess lipid, we cannot rule out the possibility that some small fraction of IAPP remains unbound and must treat estimates of helicity with caution.

### Determination of MBzP extinction coefficient

MBzP was solubilized at concentrations between 35.7 ng/mL–84.5 μg/mL in 10 mM phosphate buffer, pH 7.4. UV-Vis spectra were obtained in a 1 cm quartz cuvette on a Beckman DU-640 spectrometer from 220 nm–340 nm. Appropriate backgrounds were subtracted. Absorption at 237 nm was recorded and a best-fit linear regression was plotted according to the Beer-Lambert law: A = 6633.2C − 0.0007, R^2^ = 0.99. As the y-intercept was negligible with respect to the slope, we defined the slope of the above equation as the molar extinction coefficient. ε_237_ = 6633.2 M^−1^ cm^−1^.

### Determination of MBzP partitioning coefficient in PC bilayers

The partitioning coefficient of MBzP into PC-based bilayers was determined using previously developed methods^26^ according to equation (4)


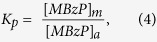


where K_p_ is the partitioning coefficient of MBzP into PC-bilayers, [MBzP]_m_ is the concentration of MBzP in the membrane phase and [MBzP]_a_ is the concentration of MBzP in solution. K_p_, expressed in measurable quantities is represented in equation (5).


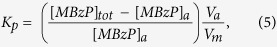


where [MBzP]_tot_ is the added MBzP concentration, V_a_ is the aqueous volume, and V_m_ is the phospholipid volume. V_m_ was approximated using a bilayer depth of 37 Å and an area per phospholipid of 69 Å^2^ 48. The relationship elaborated by equation (4) was also used to estimate the mol% MBzP based on literature values of MBzP serum levels.

Vesicles containing MBzP in these experiments were made as above except no extrusion step was used in order to ensure no loss of MBzP in the determination of soluble MBzP values. Vesicles were pelleted via centrifugation at 55,000 × g for 30 minutes and [MBzP]_a_ was determined by measuring the supernatant for absorbance at 237 nm. These data were background subtracted using the appropriate buffer. Reported K_p_ value is the average of at least three experiments performed using vesicles composed of 1, 10, 25, and 66 mol% MBzP.

### Electron Microscopy Studies

10 μL of sample were applied to carbon-coated Formvar films mounted on copper grids and the excess liquid was blotted away prior to application of 1% uranyl acetate negative sate. Specimens were imaged using a Jeol-1400 transmission electron microscope operated at 100 kV. Samples were only applied to the EM grids after enough time had elapsed for IAPP to be fully fibrilized based on ThT studies.

## Additional Information

**How to cite this article**: Okada, A. K. *et al*. Diabetic Risk Factors Promote Islet Amyloid Polypeptide Misfolding by a Common, Membrane-mediated Mechanism. *Sci. Rep.* **6**, 31094; doi: 10.1038/srep31094 (2016).

## Supplementary Material

Supplementary Information
