# Supplementary material for: Diabetic Risk Factors Promote Islet Amyloid Polypeptide Misfolding by a Common, Membrane-mediated Mechanism
Source: Sci Rep. 2016 Aug 17;6:31094. doi: 10.1038/srep31094 (PMC4987648; doi:10.1038/srep31094)
Supplement: Supplementary Information [file srep31094-s1.pdf]

Diabetic Risk Factors Promote Islet Amyloid Polypeptide Misfolding by a Common, Membrane-mediated Mechanism

Alan K. Okada[1], Kazuki Teranishi[1], J. Mario Isas[1], Sahar Bedrood[2], Robert H. Chow[3*], Ralf Langen[1*]

[1]Department of Biochemistry and Molecular Biology, Zilkha Neurogenetic Institute, University of Southern California, Los Angeles, California, USA, [2]USC Eye Institute, Department of Ophthalmology, Keck School of Medicine, University of Southern California, Los Angeles, California, USA, [3]Department of Physiology and Biophysics, Keck School of Medicine, Zilkha Neurogenetic Institute, University of Southern California, Los Angeles, California, USA

[*]Corresponding Authors: Ralf Langen, Zilkha Neurogenetic Institute, Department of Biochemistry and Molecular Biology, University of Southern California, Los Angeles, California 90033, USA. Tel.: 323-442-1323 Fax: 323-442-4404. Email: Langen@usc.edu R. H. Chow: Zilkha Neurogenetic Institute, Department of Physiology and Biophysics, Keck School of Medicine, University of Southern California. Los Angeles, CA 90089-2821, USA. Email: rchow@usc.edu

SUPPLEMENTARY FIGURES

Figure S1

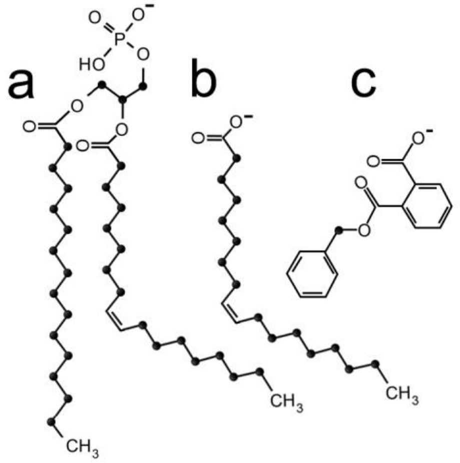

Figure S1. Cartoon schematic of the structures of A) POPA B) OA C) MBzP.

Figure S2

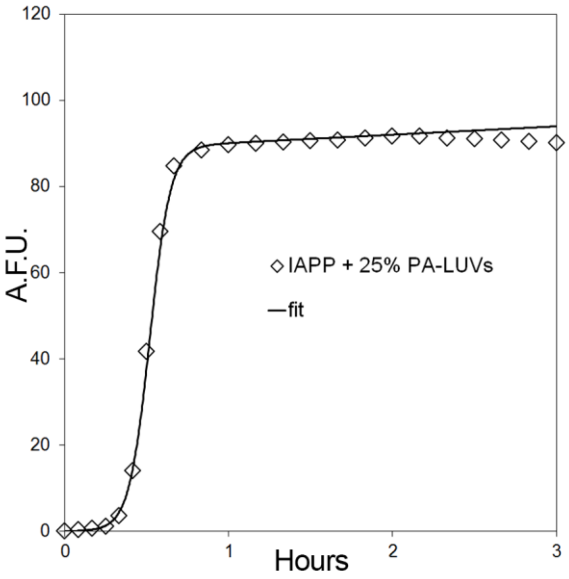

Figure S2. *Representative ThT kinetics data and associated sigmoidal fit used in the quantification of t_{50} values.* ThT fluorescence recorded during IAPP misfolding in the presence of 25 mol % PA-LUVs is shown as an example (◊). These data were fit using a sigmoidal equation (solid line), as previously described [48]. A.F.U. = Arbitrary Fluorescence Units.

Figure S3

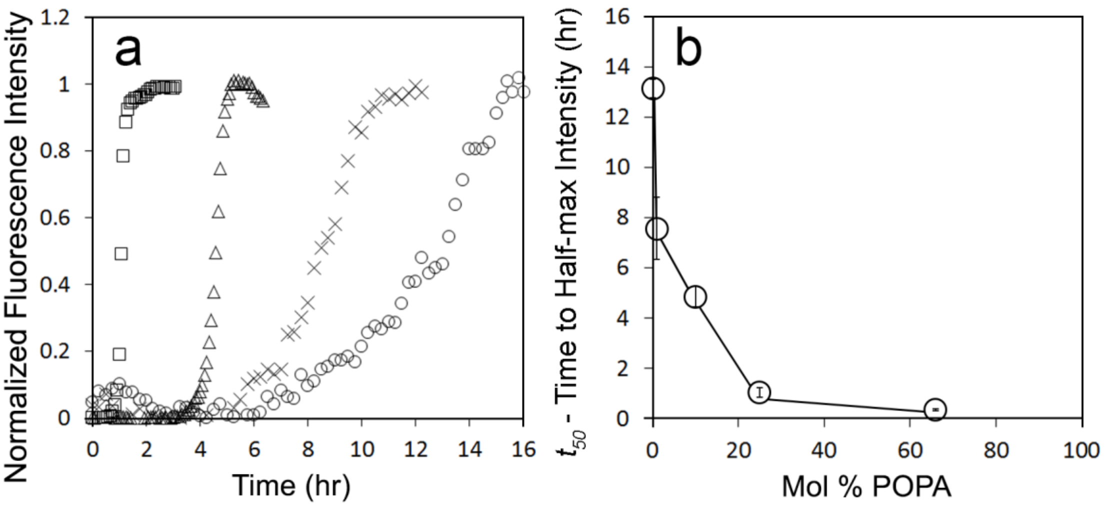

Figure S3. *Increased ionic strength right shifts the optimum PA content of vesicles for acceleration of IAPP fibrilization.* IAPP misfolding was measured as a function of time using ThT in 10mM phosphate buffer pH 7.4 with 100mM NaCl pH 7.4 during incubation with PA-LUVs. A) Representative ThT curves from experiments with 12.5μM human IAPP and 500μM composed of 66% POPA (omitted in A), 25% POPA (□), 10% POPA (Δ), 1% POPA(X), and 100% POPC (○). Normalization was performed as before (see Figure 1 or materials and methods). B) Comparison of average t_{50} values with the mol % PA from experiments in A. Error bars represent one standard deviation from a minimum of 3 experiments per condition. *($p < 0.01$, for all conditions compared with 100% POPC LUV control).*

Figure S4

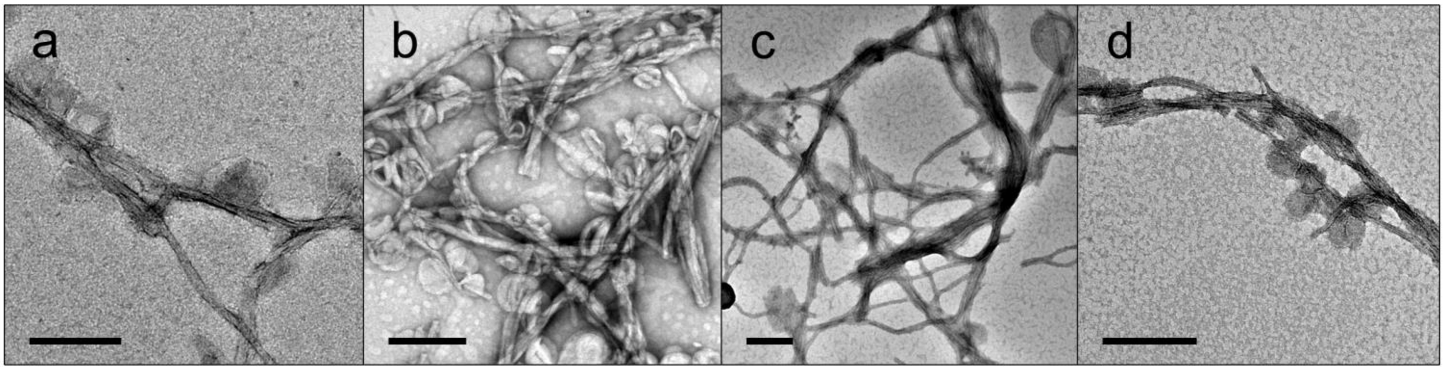

Figure S4. *Increased ionic strength does not change IAPP fibril morphology, or association with PA-LUVs by electron microscopy.* 12.5 μM IAPP was allowed to misfold in the presence of 500 μM PA-containing LUVs. Samples were incubated for the amount of time required to achieve ThT positivity before being applied to the micrograph grid. A – D) EM micrographs of IAPP in 10 mM phosphate buffer, 100 mM NaCl, pH 7.4 with 66 (a), 25 (b), 10 (c), and 1 mol% (d) PA-LUVs. Similar to low salt conditions, these images show fibrils decorated with LUVs, in some cases the circular shape of the LUVs are distorted where they contact fibrils. Lipid free sections of fibrils measure diameters of ~7 nm. Where lateral assemblies of fibrils are evident, their apparent diameters are wider than 7 nm. In addition, small spherical structures were often present and ranged from 9 nm in diameter and greater (arrows). Scale bars = 200 nm.

Figure S5

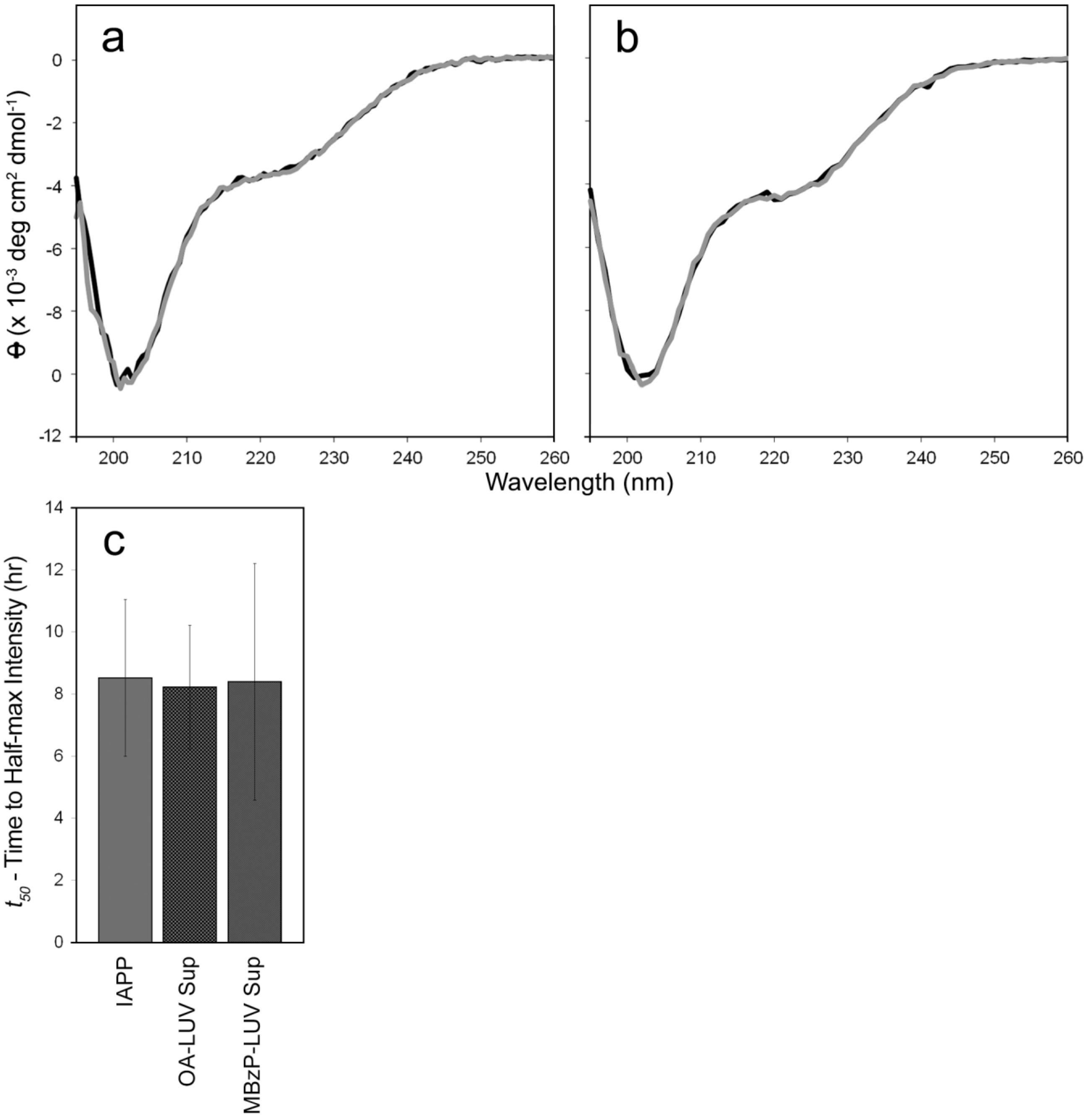

Figure S5. *Aqueous phase OA and MBzP do not accelerate IAPP aggregation.* Vehicle (phosphate buffer) or supernatants taken from 25 mol % OA- and MBzP- LUV suspensions following ultracentrifugation added to IAPP. A) CD measurements of 25 μM IAPP with supernatant (black) and with vehicle (grey) taken from OA-LUV samples. B). CD measurements of IAPP with supernatant (black) and with vehicle (grey) taken from MBzP-LUV samples. C) Average t_{50}*'s* of 12.5 μM IAPP treated with vehicle (without lipid – labelled IAPP), OA-,

or MBzP- LUV supernatants. Error bars represent 1 standard deviation from a minimum of 3 experiments. Sup = Supernatant.
